# New alleles of *C. elegans* gene *cls-2 (R107.6)*, called *xc3*, *xc4*, and *xc5*

**DOI:** 10.17912/W2RQ2X

**Published:** 2017-12-19

**Authors:** Nicholas R. Munoz, Christopher J. Black, Ethan T. Young, Diana S. Chu

**Affiliations:** 1 Department of Biology, San Francisco State University, San Francisco, California 94132, USA

**Figure 1.**
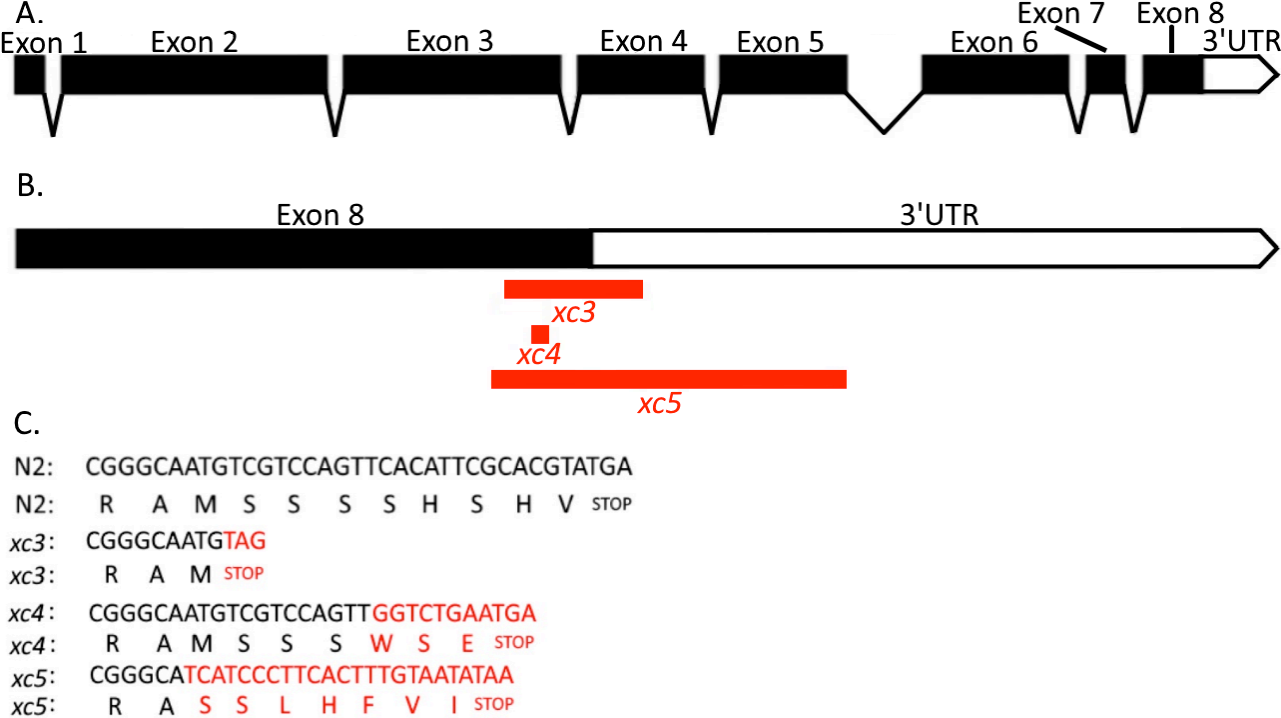
**Figure 1.** A. Map of exons, introns and the 3’UTR of *cls-2* (R107.6). B. The eighth exon and 3’UTR of *cls-2* (R107.6) with the position of the *xc3, xc4,* and *xc5* mutations indicated in red. C. Alignment of DNA and amino acid sequences in mutant and wildtype worms with mutations in red.

## Description

We have generated novel mutant alleles, named *xc3, xc4,* and *xc5,* of the gene *cls-2* (*R107.6*) that encode one of the three predicted orthologs of mammalian CLASPs and of Drosophila ORBIT/MAST, microtuble-binding proteins (Akhmanova et al., 2001; Maiato et al., 2002). In *C. elegans* CLS-2 is required for meiosis and mitosis (Cheeseman et al., 2005; Dumont et al., 2010; Espiritu et al., 2012; Maton et al., 2015; Nahaboo et al., 2015). The alleles were isolated from gene mutations generated by Non-Homologous End Joining (NHEJ) mediated repair of Cas9-generated breaks (Dickinson et al., 2013; Ran et al., 2013). The alleles were detected by PCR using the following primers, 5’- CGATACGTCGGAGCAGAGC -3’ and 5’- CGGGGGTCGAAAATCATAAGG -3’. Next Generation Sequencing allowed us to identify 30 bp flanking sequences of the alleles *xc3, xc4,* and *xc5* as TTGTCCAAGTCTACGTCAATCGGGCAATGT – [42 bp deletion] – AGCCCATAATTCCCCCGTATTCGTATCCCA, TCTACGTCAATCGGGCAATGTCGTCCAGTT – [3 bp deletion, 41 bp insertion (GGTCTGAATGACTTTCGCACTATTCCCCTATTCGCACGCCT)] – ATTCGCACGTATGATTCGTCGTTGCAATGT, and AACCTTGTCCAAGTCTACGTCAATCGGGCA – [111 bp deletion ] – TCATCCCTTCACTTTGTAATATAATTTTAT, respectively.

​Based on information about *cls-2* (*R107.6*) (WormBase, http://www.wormbase.org, WS261), the *xc3, xc4,* and *xc5* mutant alleles effect the eighth exon and the 3’-UTR in the same way in each splicing isoform (Fig.1). In the *xc3* mutant, 16 bp of the 3’UTR is deleted and a new stop codon was introduced after an 8 amino acid deletion (SSSSHSHV) of the C-terminus of the protein. In *xc4* due to an insertion causing a frameshift mutation, 5 wildtype amino acids (SHSHV) from the C-terminus will be replaced by 3 amino acids (WSE). In *xc5* the endogenous stop codon is deleted as well as 81 bp of the 3’UTR, while a new stop codon is introduced 21 bp after the mutation. Because of the deletion and new stop codon, in the *xc5* mutant 9 amino acids (MSSSSHSHV) in the C-terminus of the protein will be replaced by 7 new amino acids (SSLHFVI). Previous researchers replaced serine residues with non-phosphorylatable alanine residues to study the effect of human CLASP2 phosphorylation (Kumar et al., 2017). The mutations we have generated have multiple serine residues deleted which presents a unique opportunity to study the effect of *cls-2* (*R107.6*) phosphorylation. Since more of the 3’UTR is deleted in *xc5* than *xc3*, the 3’UTR’s function could also be studied using these mutants.

## Reagents

Alt-R® CRISPR-Cas9 crRNA Alt-R® CRISPR-Cas9 tracrRNA Alt-R® S.p. Cas9 Nuclease

Strains:**XC125** *cls-2 (xc3) unc-119 (ed3) III; ieSi38 (IV)***XC126** *cls-2 (xc4) unc-119 (ed3) III; ieSi38 (IV)***XC127** *cls-2 (xc5) unc-119 (ed3) III; ieSi38 (IV)*
